# Interdisciplinary Integration of the CVS Module and Its Effect on Faculty and Student Satisfaction as Well as Student Performance

**DOI:** 10.1186/1472-6920-12-50

**Published:** 2012-07-02

**Authors:** Nasra N Ayuob, Basem S Eldeek, Lana A Alshawa, Abdulrahman F ALsaba

**Affiliations:** 1 Medical Education Department, Faculty of Medicine, King Abdulaziz University, Jeddah, Saudi Arabia; 2 Faculty of Medicine, Mansoura University, Mansoura, Egypt; 3 Director of Cardiovascular system module, Professor of Physiology, Faculty of Medicine, King Abdulaziz University, Jeddah, Saudi Arabia; 4 Professor of Physiology, Faculty of Medicine, Ein-Shams University, Cairo, Egypt

**Keywords:** Implementation, Interdisciplinary, Curriculum, Integration, Pre-clerkship, Cardio-vascular module

## Abstract

**Background:**

Beyond the adoption of the principles of horizontal and vertical integration, significant planning and implementation of curriculum reform is needed. This study aimed to assess the effect of the interdisciplinary integrated Cardiovascular System (CVS) module on both student satisfaction and performance and comparing them to those of the temporally coordinated CVS module that was implemented in the previous year at the faculty of Medicine of the King Abdulaziz University, Saudi Arabia.

**Methods:**

This interventional study used mixed method research design to assess student and faculty satisfaction with the level of integration within the CVS module. A team from the medical education department was assembled in 2010/2011 to design a plan to improve the CVS module integration level. After delivering the developed module, both student and faculty satisfaction as well as students performance were assessed and compared to those of the previous year to provide an idea about module effectiveness.

**Results:**

Many challenges faced the medical education team during design and implementation of the developed CVS module e.g. resistance of faculty members to change, increasing the percentage of students directed learning hours from the total contact hour allotted to the module and shifting to integrated item writing in students assessment, spite of that the module achieved a significant increase in both teaching faculty and student satisfaction as well as in the module scores.

**Conclusion:**

The fully integrated CVS has yielded encouraging results that individual teachers or other medical schools who attempt to reformulate their curriculum may find valuable.

## Background

The prevailing trend in basic science curriculum changes around the world is now towards integration, both horizontally among the disciplines and vertically between basic and clinical sciences [1-4]**.** Many researchers have proven that information presented without robust cross-links and ties to clinical applications, which is tested in isolation from related subject matter, has proven difficult for students to recall after the transition to clinical clerkships [5-7].

Harvard Medical School created a hybrid curriculum in 1985 that combined problem based learning (PBL) with limited lectures and laboratories in order to help students to develop a flexible, integrated knowledge base. It demonstrated that students could learn basic science in the context of clinical medicine and humanistic care while maintaining sufficient content mastery to pass the national licensing examination with no decrement in basic science knowledge [8]**.**

Recognizing the limitations of its own traditional, discipline-based curriculum, the faculty of medicine at King Abdulaziz University (KAU) challenged both the clinical and basic science faculty members to create a new integrated curriculum to be implemented in the academic year of 2006/2007. The curriculum at KAU consists of two phases. In phase I (the pre-clinical phase) the basic sciences are taught in the form of a few core courses and system-based modules such as cardiovascular module. Phase II clinical years include the major four clerkships, in addition to some sub-specialties and a professionalism course.

Despite committee initiatives to establish integration between module content (to be temporally coordinated), student satisfaction assessed at the end of the last two academic years (2008/2009 and 2009/2010) showed incomplete satisfaction with the integration within the modules. In response to these complaints, the medical education department (MED) took a pioneering step, in association with the cardiovascular system module committee, to present an interdisciplinary integration model in the academic year of 2010/2011. This paper aimed to document the steps taken to establish such integration as well as to assess its effect on student satisfaction and performance. It will serve as a useful example for other schools aiming to improve integration levels in their curriculum.

## Methods

This interventional study used mixed method research and collected both qualitative and quantitative data to assess the effectiveness of the integrated CVS module. An ethical approval of this research article has been obtained from the biomedical research ethics committee at the Faculty of Medicine, King Abdulaziz University.

### Steps taken by the ME team

 •A focus group discussion with those faculty members participating in system based modules teaching was facilitated by MED. Ten faculty members from the basic science departments and three from the clinical departments had participated in the discussion about; their satisfaction with the integration level in the module and comments and/or complaints that had been raised by students during and at the end of all modules.

 •Reviewing the CVS module evaluations (quantitative method) filled out by the second year medical students and faculty members in the last two academic years (2008/2009 and 2009/2010) as a pilot to triangulate the results of the focus group discussion. This review revealed incomplete satisfaction of both faculty members and students with the integration within the CVS module.

 •Conducting a detailed review of the literature, looking for similar problems in the implementation of integration and the experiences and approaches taken to solve it.

 •Putting a developmental plan for enhancing integration. The CVS module was select as a model to work on because the module chairman and teaching faculty members were very enthusiastic about increasing the integration level within their module. This module is taught to the second year medical students. It has four credit hours (about 60 contact hours) distributed among lectures, practical sessions, PBL sessions, tutorials and SDL as seen in Table 1. Six disciplines; anatomy, physiology, biochemistry, pharmacology, pathology and cardiology were temporally coordinated in this module.

**Table 1:** Time (in hours) allotted to different teaching and learning methods before modification the CVS module

	**Lecture**	**Practice**	**Tutorial**	**PBL**	**Clinical presentation**	**SDL**	**Total**
Anatomy	5	3	1				
Physiology	13	2				2	
Biochemistry	4	2					
Pathology	7	2	1				
Pharmacology	4	1					
Medicine	2				2		2
Total	35	10	2	8	2	2	59

PBL: problem based learning.

SDL: student directed learning.

 •Revision of the module objectives and the objectives of each session. They correlated these objectives to the faculty program objectives. The redundant objectives were deleted while missed ones were added.

 •A theme, in the form of a clinical problem, was set for each week of the four weeks of the module. These themes were chosen with the help of the relevant clinician and according to the commonality across the module objectives and the Saudi Arabian community. The chosen themes were: heart failure, atherosclerosis and ischemic heart diseases, hypertension and arrhythmias.

 •Next came regrouping the module objectives around the chosen themes and reorganizing the teaching and learning activities (lectures, practical, tutorial, SDL and PBL sessions) to follow the themes.

 •Developing PBL cases to cover different themes.

**Figure 1:**
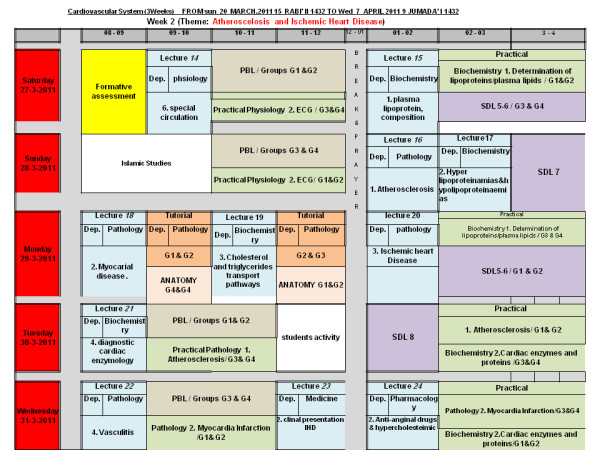
Example of a comprehensive time-table for a week of the CVS module under the theme “Atheroscelorosis and ischemic heart diseases

 •Designing a comprehensive timetable for each week Figure 1.

 •Advising teaching faculty members turned to case-based teaching of the basic sciences in order to enhance the vertical integration within the module and increased the students’ perception of the clinical relevance of basic sciences.

 •Increasing the SDL weight to occupy about 18 % of the time allotted to the module, Table 2 and developing procedures to standardize it.

**Table 2:** Time (in hours) allotted to different teaching and learning methods after modification of the CVS module

**Theme**	**week**	**Physiology**	**Anatomy**	**Pathology**	**Biochemistry**	**Pharmacology**	**Medicine**	**PBL**	**SDL**	**Total**
**Heart failure**	1^st^	L: 5 P: 1	L: 2 P: 2			L: 1	L: 1 CP:1		4	L:9 P:3 CP:1
**Arthrosclerosis/** **Ischemic Heart Disease**	2^nd^		L:1	L: 4 T: 1	L: 4 P: 2	L: 1	L: 1	4	4	L: 11 P: 2 T: 1
**Hypertension**	3^rd^	L: 5 P: 1	L: 1 T: 1	L: 1		L: 1	L: 1	4	-	L: 9 P: 1 T:1
**Arrhythmia/** **Rheumatic Heart Disease**	4^th^	L: 2		L: 2 P: 2		L: 1 P: 1	L: 1 CP:1		3	L: 6 P: 3 CP:1
**Total**	4	L: 12 P: 2	L: 4 P: 3 T:1	L: 7 P: 2 T: 1	L: 4 P: 2	L: 4 P: 1	L: 4	8	11	L: 35 P:10 T:2 CP:2

L: Lecture.

P: Practical.

T: Tutorial.

CP: Clinical Presentation.

 •Regarding the assessment, a workshop to train faculty members on constructing integrated items was held by the ME team. The assessment tools presented in the workshop included problem-based questions (PBQ), modified essay questions (MEQs) and objective structured practical examination (OSPE). These tools were used intentionally to assess the higher levels of cognition such as application and evaluation of knowledge. This was accomplished after the development of an exam blueprint through the collaboration between the ME team and the CVS module members.

 •Highlighting the importance of formative assessment through demonstrating its role in providing students with feedback on their performance, helping them detecting their gaps of knowledge, planning to fill these gaps and familiarizing them with the newly used assessment tools before the final summative exam.

 •Maintaining a close monitoring and follow up process to ensure proper implementation of what has been planned. This was accomplished formally through direct contact with the head of the CVS module.

 •To measure the effectiveness of the developed module, both faculty and student satisfaction was measured via module evaluation questionnaires. The results were compared with results measured in the academic year of 2009/2010. Student assessment results, in the form of overall success rate and the percentage of students grades as well as the item analysis results of the CVS exams for the two consecutive years (2009/2010) and (2010/2011), were also compared.

 •Statistical analysis was undertaken using a statistical package of social science (SPSS) software, version 16 (2005). The qualitative data were presented in the form of number and percentage. A chi-square test, with linear trends, was used for ordinal qualitative data. A percentage rate for teaching faculty member satisfaction was calculated for the module evaluation. Significance was considered at p value less than 0.05.

## Results

Focus group discussion with the faculty members participating in system based modules in the years from 2008–2010 revealed incomplete satisfaction which the integration level in their particular modules.

In spite of the initial resistance among some faculty members towards the developed integrated CVS module during its preparatory phase, the result of focus group discussion, after implementation of the developed module, revealed an increase in satisfaction with the module. They even sought help from ME team members to implement integration in other modules in which they were involved.

The result of the course evaluation questionnaire completed by students showed that overall student satisfaction was 53.88 % in 2009/2010 and 72.65 in 2010/2011. There was a highly significant increase in student satisfaction regarding CVS module contents in 2010/2011 compared to the previous year, see Table 3. When it came to satisfaction with the module teaching faculty members, there was a significant increase in overall student satisfaction in 2010/2011, see Table 3. It was noted that there was a significant increase in the overall student satisfaction with the CVS assessment plan in 2010/2011, see Table 4. On the other hand, there was an insignificant increase in overall student satisfaction with the educational resources provided in the CVS in 2010/2011when compared to the previous year, see Table 4.

**Table 3:** Results of students responses to course evaluation questionnaire (in relation to module contents and faculty members) of the CVS module in (2009/2010) and (2010/2011)

**Items of comparison**	**2009/2010 Satisfaction N=129 %**	**2010/2011 Satisfaction N=152 %**	**Test of significance**
**Module contents**			
Objectives of course are clear	69.5	93	P<0.001***
Objectives of each teaching formats were explained at its start	69	85	P=0.002**
The modules contents show relation between basic & clinical sciences	81.1	89	P=0.089
The practical part is related to the theoretical part	67.4	87	P<0.001***
This course help to develop my self-learning capacity	63.3	86	P<0.001***
PBL sessions improve my learning achievements	61.7	80	P=0.001**
This course is good & useful for my future career	80.9	94	P<0.001***
Overall satisfaction	70.5	87.5	P<0.01**
**Faculty members**			
address the contents of the module as stated study guide	64	82	P=0.002**
encourage students to ask questions during teaching	37.7	74	P<0.001***
make good use of different types of educational methods	46.1	73	P<0.001***
Faculty members use clinical cases in teaching	64.1	94	P<0.001***
clearly explain the methods of assessment from the start of the course	46.1	84	P<0.001***
Faculty members provide positive feedback after each assessment	34.2	70	P<0.001***
encourage students for self-directed learning	50.8	77	P<0.001***
encourage students for electronic learning	50.8	77	P<0.001***
usually available in their office hours	23.2	50	P<0.001***
Overall satisfaction	46.5	75.6	P<0.001***

**Table 4:** Results of students response to the course evaluation questionnaire (in relation to assessment and educational resources) of the CVS module in (2009/2010) and (2010/2011)

**Items of comparison**	**2009/2010 Satisfaction N=129 %**	**2010/2011 Satisfaction N=152 %**	**Test of significance**
**Assessment**			
Assessment methods were fair	41.7	75	P<0.001***
Assessment reflects what was taught.	43.6	84	P<0.001***
Assessment method challenge students more than memorize.	50	85	P<0.001***
Number of tests was reasonable for the course.	55.4	76	P=0.001**
These is Formative assessment	37.9	75	P<0.001***
The time of formative assessment is suitable.	37.9	75	P<0.001***
Overall satisfaction	44.5	65.8	P<0.001***
**Educational resources**			
Educational materials were available.	66.7	67	P=0.96
Educational materials posted of EMES MED (education management of electronic system for Medicine) at earlier time.	39.3	50	P=0.084
I frequently use the library to search learning material.	39.3	69	P<0.001***
The library contains adequate number of books for the course.	45.3	72	P<0.001***
Lecture rooms are well-equipped.	45.3	62	P=0.006**
Laboratories are well-equipped	53.2	64	P=0.084
Rooms for PBL are well constructed	53.2	51	P=0.81
Student’s study guide helped me much throughout the course.	35.5	59	P<0.001***
Overall satisfaction	54	61.7	P=0.23

Though the overall success rate was higher (100 %) during the academic year of 2009/2010 when compared to that of 2010/2011 (98.6 %), the percentage of students who received an A grade was significantly higher in (2010/2011). On the other hand, the percentage of students who got a D grade was significantly lower in (2010/2011) see Table 5.

**Table 5:** Final scores of the students in final CVS module exam in (2009/2010) and (2010/2011)

**Grade**	**2009/2010 N= 344 N %**	**2010/2011 N= 379 N %**	**Test of significance**
A	26 (7.5)	85 (22.4)	Chi-square test= 37.29 P<0.001***
B	94 (27.3)	123 (23.4)	
C	94 (27.3)	83 (21.8)	
D	130 (37.7)	53 (13.9)	
F	0 (0)	5 (1.3)	
Overall success rate	344 (100)	374 (98.6)	

Item analysis of the CVS exams for the two consecutive years (2009/2010 and 2010/2011) was carried out. Regarding the difficulty index, the results showed that the majority of the exam items given in 2009/2010 were very easy (36.6 %) and easy (50 %) items. After implementation of the developed integrated module, the percentages of the very easy and easy items had been reduced to 11.7 % and 48.3 % respectively. The percentage of the excellent items increased from 14.4 in 2009/2010 to 36.7 in 2010/2011, see Table 6.

**Table 6:** Difficulty and Discrimination indexes of the items of the CVS module exam in (2009/2010) and (2010/2011)

	**2009/2010N= 60N %**	**2010/2011N= 60N %**	**Test of significance**
**Difficulty index**			
Very easy (Conditionally acceptance) (0.9-1)	22 (36.6)	7 (11.7)	Chi-square test with liner trends X^2^= 16.31 P=0.009**
Easy (0.70-0.9)	30 (50)	29 (48.3)	
Excellent (0.3-0.7)	8 (14.4)	22 (36.7)	
Moderate (0.15-0.30)	-	2 (3.3)	
Too difficult (≤0.15)	-	-	
**Discrimination index**			
Very good item (≥ 0.30)	8 (13.3)	42 (70)	Chi-square test with liner trends X^2^= 44.6 P<0.001**
Reasonably good (0.20-0.29)	29 (48.3)	8 (13.3)	
Marginal item (0.09-0.20)	11 (18.3)	9 (15)	
Poor (zero)	10 (16.6)	1 (1.7)	
Unaccepted discrimination (Negative)	2 (3.3)	-	

Regarding the discrimination index, the results showed that the percentage of the very good discriminating items increased from 13.3 % in 2009/2010 to 70 % in 2010/2011. On the other hand, the percentage of the poor discriminating items, the items that need to be rejected, decreased from 16.6 % in 2009/2010 to 1.7 % in 2010/2011, see Table 6.

Because of the small number of faculty members involved in the CVS module teaching, significance tests could not be applied to the module evaluation questionnaire completed by them. Instead, the percent of change was calculated for each assessed item. The results showed that there was a 51 % increase in the overall faculty member satisfaction with the module in 2010/2011. The percentage of increase in the satisfaction index with all of the assessed items did not exceed 50 %, except in five items. These include: encouraging SDL, building student analytical and problem solving skills, implementing PBL successfully, availability of learning resources and alignment between the course objectives, instruction and assessment Table 7.

**Table 7:** Results of course evaluation questionnaire by teaching faculty of the CVS module in (2009/2010) and (2010/2011)

**Items of comparison**	**2009/2010 Satisfaction Index**	**2010/2011 Satisfaction Index**	**Percentage of change**
Integration is implemented during instruction	60	88	46
Learning objectives were made clear to students from the start	72	88	22
Learning objectives were made clear to faculty members from the start	70	80	17
Course encourages SDL	55	84	52
Course encourage group work	62	80	29
Course build students analytical and problem solving skills	55	84	52
PBL was successfully implemented as planned	60	92	53
Learning resources available helping in implementing the course objectives	45	88	95
Course web pages were relevant to course objectives	42	58	38
Number of staff members is sufficient in relation to tasks of the course	60	86	43
There is alignment between the course objectives, instruction and assessment	46	84	82
Assessment is based on a blueprint.	40	60	33
Various methods of assessment are used	55	70	30
Formative assessment without scores are used	60	84	38
Early feedback is provided to students	61	84	37
There is continuous and final summative assessment	70	90	28
The course team utilizes the results of interpretation of item analysis	55	82	49
Overall statistical index	53	80	51

## Discussion

Medical and dental education curricula are continually developing by incorporating advancements, such as horizontal and vertical integration, to address the contemporary needs of their students [9]**.**

Curriculum integration enables learners to recognize how diverse concepts and/or processes interrelate [10]**.** This concept has received much attention across the health sciences [9,11]**.** The clear build-up of the curriculum and the vertical and horizontal integration of subject knowledge seem to have significantly reduced the lack of regulation [12]**.** As a result, the faculty of medicine at KAU was encouraged to launch an integrated, system- based curriculum to be delivered to students during Phase I (pre-clerkship years).

Since the planned and delivered curriculum can be significantly different, the first question which came to the authors’ mind was, ‘How much does the delivered curriculum differ from the planned integrated curriculum? [13]**, t**o answer this question, the ME team investigated both student and faculty member satisfaction with the integrated curriculum through both qualitative and quantitative methods.

On designing the newly developed interdisciplinary, integrated CVS module, the tips described by **Malik and Malik** were beneficial. They described how integration can be enhanced from harmonization to interdisciplinary integrated level by avoiding commonly committed mistakes [14].

The overall satisfaction of the students after implementation of the integrated CVS module in 2010/2011 was 72.65 %. It seemed to be slightly lower than the overall student satisfaction rate (77.63 %) that was recorded by **Mehr et al**. The latter results were obtained after assessing an elective integrated training module of the brain’s basal ganglia which was designed and implemented by a multidisciplinary team [15]**.**

This study revealed a significant increase in overall student satisfaction (from 53.88 % to 72.65 %) after implementation of the interdisciplinary integrated module. These results seemed to be in agreement with the results obtained by Klement et al. while they reported Morehouse School of Medicine experience in integrating its first year medical curriculum in 2005. The integration process was expanded to include first year basic science courses (Human Morphology, Biochemistry, Physiology, and Neurobiology). The outcomes of the restructured curriculum include higher or equivalent subject examination average scores, enhanced student satisfaction [16].

These results were in contradict with the findings of Harvard medical school in 2011 when it replaced its dedicated Preventive Medicine and Nutrition course with an integrated curriculum and assessed student satisfaction with both of them. It was found that students with the integrated curriculum were less satisfied with both the quantity and quality of their nutrition education [17]**.** This decreased satisfaction could be attributed to the reduced content of the integrated curriculum that did not satisfy student curiosity. That was not the case in this study as a sufficient amount of content was secured in the integrated CVS module.

It was found that the overall success rate was lower in 2010/2011 than in 2009/2010 (98.6 % and 100 % respectively). This might indicate that the exams of the integrated module were more difficult and discriminating and signified a more effective assessment plan.

Implementing case-based teaching seemed to be one of the causes that elicited an increase in student satisfaction in this study and was described by previous ones [18]**.** Although the ME team failed to convince the CVS teaching faculty to reduce the number of lectures, student satisfaction was not compromised but actually increased. The finding was in accordance with other researches [19]**.**

Fostering SDL was among the methods that the ME team stressed during the integration elements as its role was emphasized by previous studies [20,21]. Weekly, online, formative assessment (quizzes) using the electronic system was another method which was introduced to provide regular feedback to students on their learning process. This was advised by **Wilkerson et al**. and had proven effective [22]**.**

Among the challenges facing the ME team was the inherent resistance of some faculty member to any change. Regular weekly meetings with the CVS module committee were sufficient to convince and motivate them. It was challenging to make SDL standardized as faculty members were conducting it in different ways and some of them did not assess it at all. The team put together standardized regulations for SDL, starting from setting its topics, learning objectives and assessment method and providing feedback on student learning. Increasing the weight of the SDL (percentage of its hours from the total contact hour allotted to the module) was another challenge that faced the team. This was because faculty members had strong beliefs in the effectiveness of the teacher centered approach and did not rely on SDL as an active and effective approach to student learning.

It was challenging to convince the faculty members of different disciplines to share constructing test integrated items that tackle different disciplines on assessment. They believed that the item construction process should be a confidential and individual process that should not be shared with others. The ME team had conducted workshops to train them and succeeded to convince them to do so after demonstrating the educational impact of integrated assessment on student learning.

## Conclusion

Since the prevailing trend in basic science curriculum changes is now towards integration, demonstrating this experience of upgrading the integration level within the pre-clerkship curriculum and documenting its effectiveness could be helpful for medical schools that are willing to enhance integration levels in their curriculum. It could also prove useful to those who intend to integrate their conventional discipline-based curriculum in order to help their students to cross-link and tie information to clinical applications and recall it after the transition to clinical clerkships.

## Competing interests

The authors report no declarations of interest.

## Authors' contributions

NNA shared in research conception and design, acquisition of data, interpretation of data in addition to writing the manuscript and revising it critically and final approval of the version to be published. BSE shared in research conception and design, acquisition of data, analysis of data in addition to revising manuscript critically and final approval of the version to be published. LAA shared in research conception and design, writing the manuscript and revising it critically and final approval of the version to be published. AFA shared acquisition of data, revising the manuscript critically and final approval of the version to be published. All authors read and approved the final manuscript.

## Authors’ information

NASRA N. AYUOB is assistant professor in the Medical Education Department, faculty of medicine. She is particularly interested in curriculum designing and integration. She is one of the members of the MED who are responsible for providing educational services to the basic science departments. She is a physician. She had a master and MD in the microscopic Anatomy in addition to a joint master in Medical education JMHPE for Maastricht.

BASEM S. ELDEEK is associate professor in the Medical Education Department, faculty of medicine. He is particularly interested in medical education research. He is one of the members of the MED who are responsible for providing educational services to the clinical departments. She is a physician. He had a master and MD in the public health in addition to a joint master in Medical education JMHPE for Maastricht.

LANA A. ALSHAWA is assistant professor in the Medical Education Department, faculty of medicine. She is particularly interested in PBL. She is one of the members of the MED who are responsible for providing educational services to the basic science departments. She is a dentist. She had a PhD in PBL.

ABDULRAHMAN F. ALSABA is professor of physiology, faculty of medicine. He is the head of the CVS module committee.

## Pre-publication history

The pre-publication history for this paper can be accessed here:

http://www.biomedcentral.com/1472-6920/12/50/prepub
